# *OsEXPA7* Encoding an Expansin Affects Grain Size and Quality Traits in Rice (*Oryza sativa* L.)

**DOI:** 10.1186/s12284-024-00715-x

**Published:** 2024-05-23

**Authors:** Xinwei Zhang, Ying Wang, Mingyu Liu, Peiwen Yan, Fuan Niu, Fuying Ma, Jian Hu, Shicong He, Jinhao Cui, Xinyu Yuan, Jinshui Yang, Liming Cao, Xiaojin Luo

**Affiliations:** 1 State Key Laboratory of Genetic Engineering and Engineering Research Center of Gene Technology (Ministry of Education), School of Life Sciences, Fudan University, Shanghai, China; 2 Ministry of Education Key Laboratory for Biodiversity Science and Ecological Engineering, Department of Ecology and Evolutionary Biology, School of Life Sciences, Fudan University, Shanghai, China; 3 Institute of Crop Breeding and Cultivation, Shanghai Academy of Agricultural Sciences, Shanghai, China; 4 Ministry of Education, Key Laboratory of Crop Physiology, Ecology and Genetic Breeding College of Agronomy, Jiangxi Agricultural University, Nanchang, China

**Keywords:** Expansin, *OsEXPA7*, Plant hormone, Grain size, Yield traits, Rice quality

## Abstract

**Background:**

Yield and quality are the two most important traits in crop breeding. Exploring the regulatory mechanisms that affect both yield and quality traits is of great significance for understanding the molecular genetic networks controlling these key crop attributes. Expansins are cell wall loosening proteins that play important roles in regulating rice grain size.

**Results:**

We investigated the effect of *OsEXPA7*, encoding an expansin, on rice grain size and quality. *OsEXPA7* overexpression resulted in increased plant height, panicle length, grain length, and thousand-grain weight in rice. *OsEXPA7* overexpression also affected gel consistency and amylose content in rice grains, thus affecting rice quality. Subcellular localization and tissue expression analyses showed that *OsEXPA7* is localized on the cell wall and is highly expressed in the panicle. Hormone treatment experiments revealed that *OsEXPA7* expression mainly responds to methyl jasmonate, brassinolide, and gibberellin. Transcriptome analysis and RT-qPCR experiments showed that overexpression of *OsEXPA7* affects the expression of *OsJAZs* in the jasmonic acid pathway and *BZR1* and *GE* in the brassinosteroid pathway. In addition, *OsEXPA7* regulates the expression of key quantitative trait loci related to yield traits, as well as regulates the expression levels of *BIP1* and *bZIP50* involved in the seed storage protein biosynthesis pathway.

**Conclusions:**

These results reveal that *OsEXPA7* positively regulates rice yield traits and negatively regulates grain quality traits by involving plant hormone pathways and other trait-related pathway genes. These findings increase our understanding of the potential mechanism of expansins in regulating rice yield and quality traits and will be useful for breeding high-yielding and high-quality rice cultivars.

**Supplementary Information:**

The online version contains supplementary material available at 10.1186/s12284-024-00715-x.

## Introduction

Rice is one of the most important food crops, and is the main food for more than half of the world’s population (Yuan 2015). Increasing rice yields is the key to solving the problem of food shortages due to rapid global population growth, a reduction in the arable land area, and global warming (McClung 2014). The yield of rice depends mainly on the number of panicles, the number of grains in a panicle, and the grain weight (Lan et al. 2023). Grain weight depends on grain size and the grain-filling degree, while grain size is mainly determined by grain type, including grain length, width, and thickness (Ling et al. 2023; Hu et al. 2012). There is a direct relationship between grain size and rice yields. However, grain size not only affects the yield, but also the appearance quality of grains (Sakamoto et al. 2008; Harberd et al. 2015; Dong et al. 2021). The quality of rice is mainly determined by the synthesis, composition, distribution, and accumulation of various nutritional components and storage products, and is reflected by internal and external attributes such as appearance, taste, and flavor (Zhou et al. 2013; Custodio et al. 2019; Sreenivasulu et al. 2022). Therefore, the development of high-yielding and high-quality rice cultivars is beneficial to meet human demands for rice yield and nutrition.

Rice seed is composed of the glume, endosperm, and embryo, among which the glume plays a decisive role in grain size (Li et al. 2016). At the early developmental stage, cells in the glume divide to increase the cell number, and then the cells begin to expand and increase in size. The final number and size of cells in the glume determine the grain length, grain width, and grain thickness, thus affecting the grain size (Li et al. 2018). Cells change size by expanding, mainly as a result of the actions of expansins in the cell wall. Previous studies have reported that expansins break the hydrogen bonds connecting cellulose and hemicellulose, thereby loosening the cell wall. This allows the cell wall polymer to slide, which allows the cell to expand (Bashline et al. 2014; Fukuda et al. 2014). However, the details of how expansins function, and their range of activity, have not yet been fully confirmed. In addition, grain size is affected by changes in the number and size of cells mediated by several plant hormones and signaling pathways, including jasmonic acid (JA) (Hu et al. 2021), the auxin signaling pathway (Ma et al. 2023), gibberellin (Hu et al. 2018a, b), brassinolide signaling (Sun et al. 2021), abscisic acid (Cheng et al. 2014), and cytokinin (Xiao et al. 2019). Expansins are also able to regulate plant hormones. However, there is still much to learn about the effects of expansins on grain size via their effects on hormones.

Expansins were first identified in cucumber hypocotyls, they loosened plant cell walls in a non-enzymatic but pH-dependent manner (Choi et al. 2003). Based on their sequence and biochemical activity, expansins can be divided into four categories: α-expansin (EXPA) β-expansin (EXPB), expansin like A (EXLA) and expansin like B (EXLB). These four groups show very low sequence similarity (20–25%), but their structures are highly conserved (Kende et al. 2004). Expansins have two structural domains: domain I and domain II. Domain I is usually considered as the catalytic center, homologous to that of glycoside hydrolase family 45 (GH45) proteins, but it lacks catalytic activity. Domain II was reported to be homologous to grass pollen allergens in maize (Sampedro et al. 2005). Expansins mainly play roles in seed germination (Yan et al. 2014), root formation (Zou et al. 2015), plant vegetative growth (Choi et al. 2003), reproductive growth, fruit enlargement and maturity (Brummell et al. 1999; Hiwasa et al. 2010), and responses to abiotic stress. However, there are no reports on the effects of expansins on rice quality. Studies exploring such roles of expansins will provide new information about the regulation of rice quality and about the diverse functions of these proteins.

Previous studies have shown that RNA-binding protein-A (RBP-A) encoded by *GL11* (*LOC_Os11g41890*), and *OsEXPA7* (LOC_Os03g60720) are downstream targets of *GL11* (Ren et al. 2023). *GL11* can negatively regulate grain size and thousand-grain weight in rice, while the effect of *EXPA7* on rice grain needs to be further explored. In this study, we found that *OsEXPA7* regulates the expression levels of *OsJAZs* in the JA pathway, as well as key quantitative trait loci (QTLs) and genes involved in the seed storage protein biosynthesis pathway, thus affecting rice yield and quality traits. Our results show that *OsEXPA7* positively regulates rice grain size and thousand-grain weight, but negatively regulates rice quality. The information obtained in this study expands the known functional mechanisms of the expansin OsEXPA7 in terms of its contribution to rice yield and quality traits, and establishes a further link between rice yield and quality. Ultimately, our findings will be useful for further research to generate high-yielding and high-quality rice varieties.

## Results

### *OsEXPA7* Positively Regulates Grain Size and Negatively Regulates Rice Quality

**Fig. 1:**
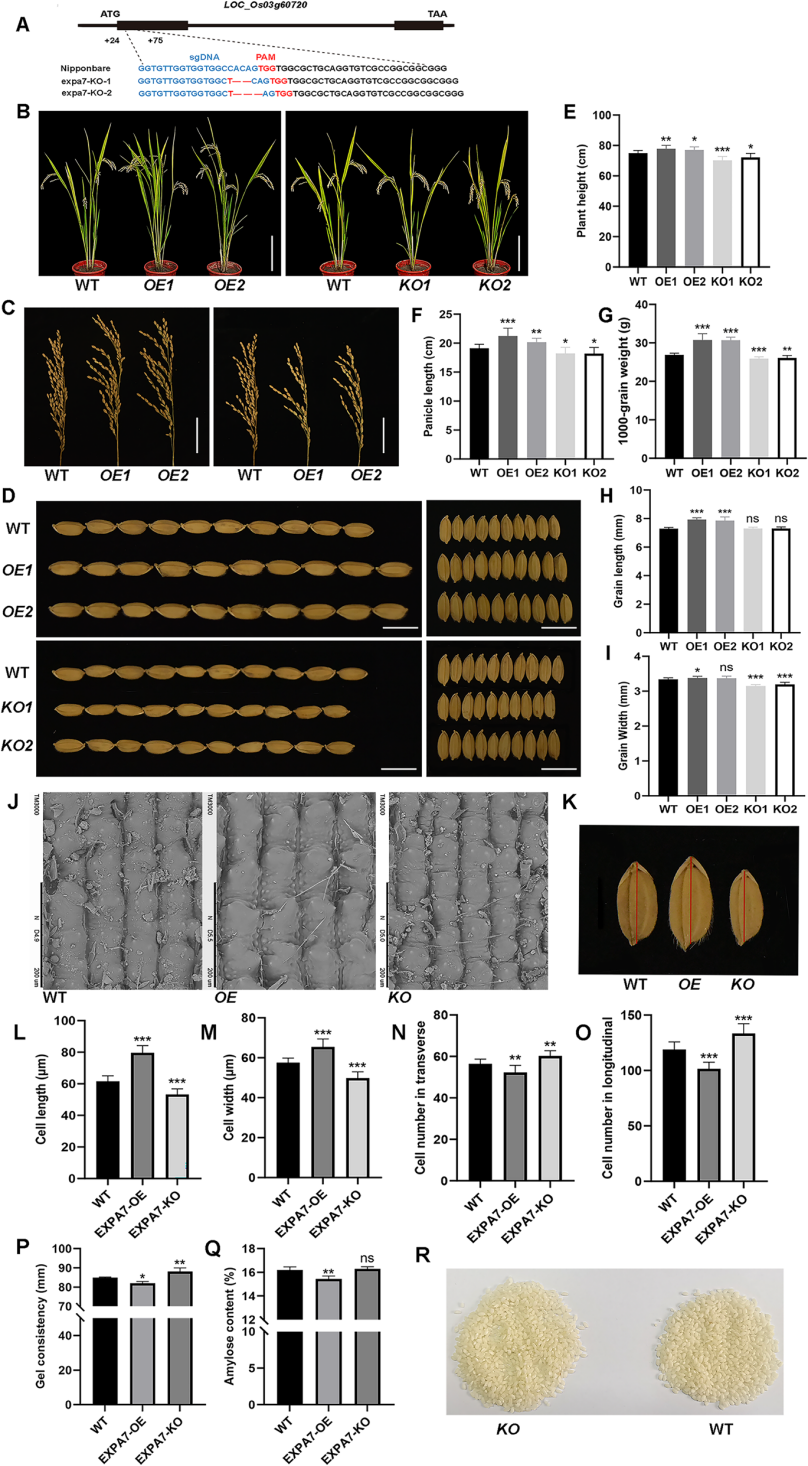
Phenotypes of *OsEXPA7* transgenic lines. **A** The knockout type of *OsEXPA7*. **B**–**D**. Phenotypes (plant height, panicle length, and grain) of wild-type and *OsEXPA7* overexpression (OE) and knockout (KO) lines. **E**–**I**. Growth traits (plant height, panicle length, thousand-grain weight, grain length, and grain width) of wild-type and *OsEXPA7* OE and KO lines (*n* = 10). **J**. Scanning electron micrographs of grains of wild-type, *OsEXPA7* OE, and *OsEXPA7* KO lines. **K**. Grain phenotype of wild-type, *OsEXPA7* OE, and *OsEXPA7* KO lines, red solid lines indicate longitudinal direction. **L**–**O**. Cell length, cell width, cell numbers in transverse and longitudinal directions in wild-type, *OsEXPA7* OE, and *OsEXPA7* KO lines (*n* = 10). **P**–**Q**. Rice grain quality (gel consistency and amylose content) of wild-type, *OsEXPA7* OE, and *OsEXPA7* KO lines (*n* = 3). **R** Rice grain phenotypes of wild-type and *OsEXPA7* KO lines

To elucidate the role of *OsEXPA7* in rice growth, we used the *CRISPR/Cas9* system to create two *OsEXPA7* (LOC_Os03g60720) knock-out lines of Nipponbare, *KO-1* and *KO-2*. The mutation sites are shown in Fig. 1A. We also produced two transgenic lines overexpressing *OsEXPA7*, designated as *OE*-1 and *OE*-2. The phenotype analysis results showed that, compared with the control, the KO lines had smaller plant height and shorter panicle length, and the OE lines had greater plant height and longer panicle length (Fig. 1E–F). These results indicate that *OsEXPA7* positively regulates rice plant height and panicle length. Interestingly, the KO lines showed a decrease in grain width and thousand-grain weight, but no significant change in grain length (Fig. 1G–I), while the OE lines showed an increase in grain length and thousand-grain weight but no significant change in grain width (Fig. 1G–I). These results indicate that *OsEXPA7* is involved in the regulation of grain size and yield traits.

To further explore the effect of *OsEXPA7* on grain size, we measured the size of the outer epidermis cells of mature rice grain glumes. Compared with the control group, the OE lines of *OsEXPA7* showed increased cell length and width (Fig. 1J–M), while the KO lines of *OsEXPA7* showed decreased cell length and width (Fig. 1J–M). However, in terms of cell numbers, both horizontally and vertically, the number of cells was decreased in the OE lines but increased in the KO lines (Fig. 1N–O). This result indicates that *OsEXPA7* positively regulates grain cell size and but negatively regulates cell number, thus affecting rice grain size and yield traits.

The amylose content (AC), gel consistency (GC), and pasting temperature (PaT) are the three main indicators of rice quality (Zhang et al. 2020). Among them, AC is a major determinant of palatability, viscosity, clarity, and digestibility of rice grains (Hu et al. 2023). To verify whether *OsEXPA7* regulates rice quality, we performed rice quality tests on the wild-type, OE, and KO lines. Compared with the wild-type rice grains, those of the OE lines showed decreased GC, and those of the KO lines showed increased GC (Fig. 1P). Compared with the wild-type rice grains, those of the OE lines showed significantly decreased AC, while those of the KO lines showed no significant change in AC (Fig. 1Q). In terms of rice grain appearance, the KO lines performed better than the wild-type (Fig. 1R). There was no difference in PaT or chalkiness among the wild-type, KO, and OE lines (Table 1). These findings indicate that *OsEXPA7* negatively regulates rice quality by affecting AC and GC.

**Table 1:** Grain quality of wild-type and *OsEXPA7* transgenic lines

Sample	Amylose content (%)	Gel consistency (mm)	Pasting Temp (℃)	Chalkiness (%)
Wild-type	16.20 ± 0.22	85 ± 0.20	74.75 ± 0.15	4.20 ± 0.24
*OsEXPA7* KO	16.28 ± 0.16	88 ± 1.55*	73.85 ± 0.24	3.75 ± 0.10
*OsEXPA7* OE	15.43 ± 0.21*	82 ± 0.82**	73 ± 0.18	4.35 ± 0.20

Data represent the mean ± SEM (Standard error of the mean) of three replicates. *P < 0.05, **P < 0.01

### Subcellular Localization and Tissue Expression of *OsEXPA7*

**Fig. 2:**
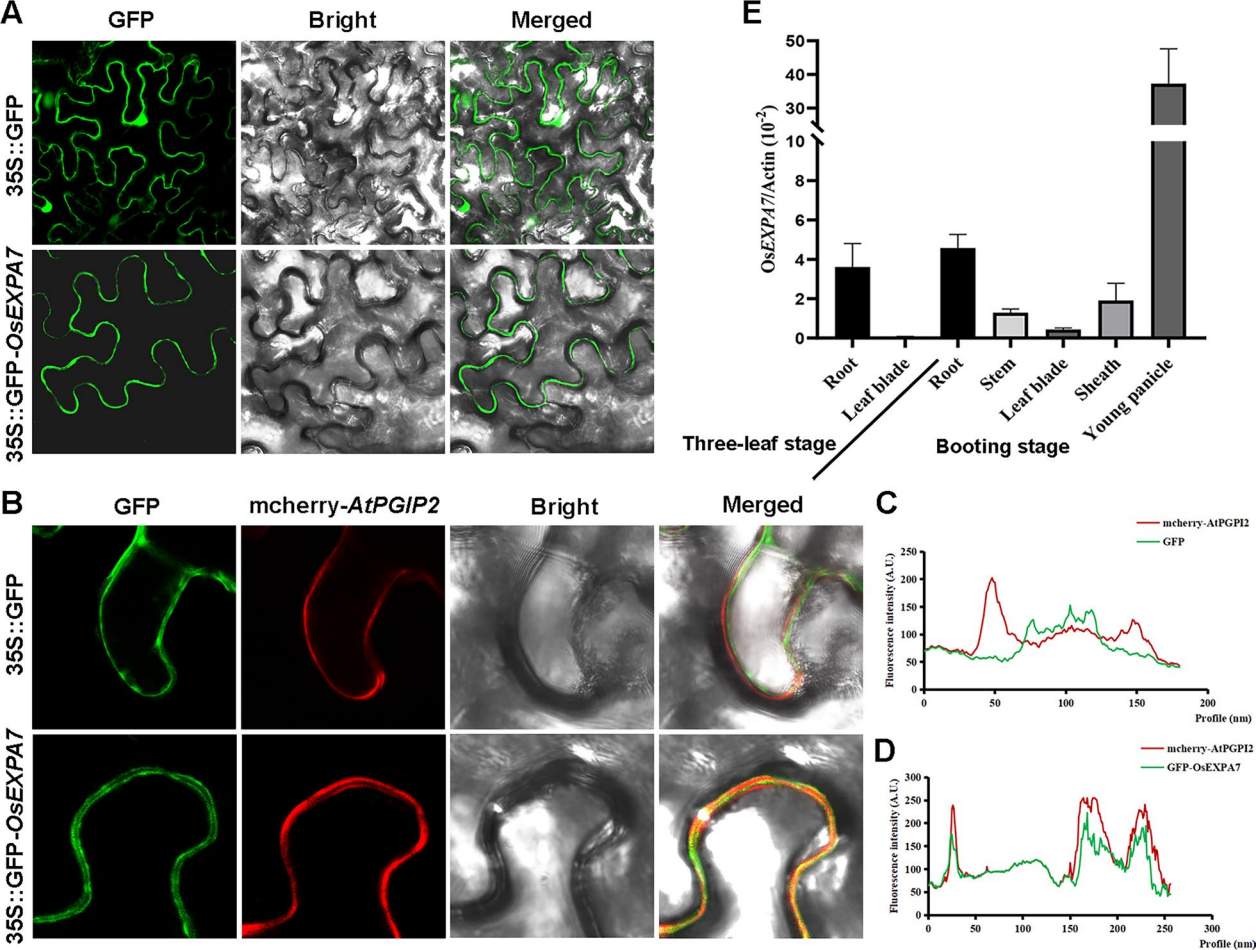
Subcellular localization and expression pattern of *OsEXPA7.* **A** Subcellular localization of *OsEXPA7*. **B** Colocalization of *OsEXPA7* and AtPGIP2*.* **C**, **D** Colocalization analysis of mean fluorescence intensity. **E** Transcript profiles of *OsEXPA7* in rice plants at the three-leaf stage and booting stage (*n* = 3)

Subcellular localization analyses using the online software Cell Ploc (http://www.csbio.sjtu.edu.cn/bioinf/Cell-PLoc/) predicted that *OsEXPA7* mainly localizes on the cell wall. To verify this result, we constructed the 35S::GFP-*OsEXPA7* vector and the 35S::mcherry-*AtPGIP2* vector (as a cell wall marker) and transiently expressed the fusion proteins of 35S::GFP-*OsEXPA7* and 35S::mcherry-*AtPGIP2* in tobacco leaves. AtPGIP2 is a known cell wall protein (De Caroli et al. 2020). The subcellular localization of *OsEXPA7* was detected by laser confocal microscopy. The results show that the fluorescence signal of the empty GFP vector did not overlap with the red fluorescence signal of mcherry-*AtPGIP2*, while the green fluorescence signal of GFP-*OsEXPA7* overlapped with the red fluorescence signal of mcherry-*AtPGIP2* (Fig. 2A, B). To further verify whether the fluorescence signals were co-localized or not, we performed co-localization analysis using the Plot Profile function of ImageJ. In the control group, the GFP empty green fluorescence signal curve did not coincide with the peak position of the red fluorescence curve of mcherry-*AtPGIP2* (Fig. 2C). In the experimental group, the peak position of the green fluorescence signal curve of GFP-*OsEXPA7* was the same as that of the red fluorescence curve of mcherry-*AtPGIP2* (Fig. 2D), indicating that they had the same subcellular localization. These results confirm that *OsEXPA7* is localized on the cell wall, consistent with its role in regulating grain size.

To further explore how *OsEXPA7* participates in plant development, its transcript profiles in different tissues were determined. Samples of roots, leaf blades, stems, sheaths, and young panicles were collected from wild-type rice at the three-leaf stage and the booting stage, total RNA was extracted, and then the expression levels of *OsEXPA7* were determined by quantitative reverse transcription PCR (qRT-PCR). The results showed that *OsEXPA7* was mainly expressed in the roots at the three-leaf stage. At the booting stage, *OsEXPA7* was mainly expressed in the roots and young panicles, with particularly high expression levels in the panicles (Fig. 2E). This result suggests that *OsEXPA7* plays a key role in regulating panicle development and grain size.

### *OsEXPA7* Responds to Plant Hormone Treatments

**Fig. 3:**
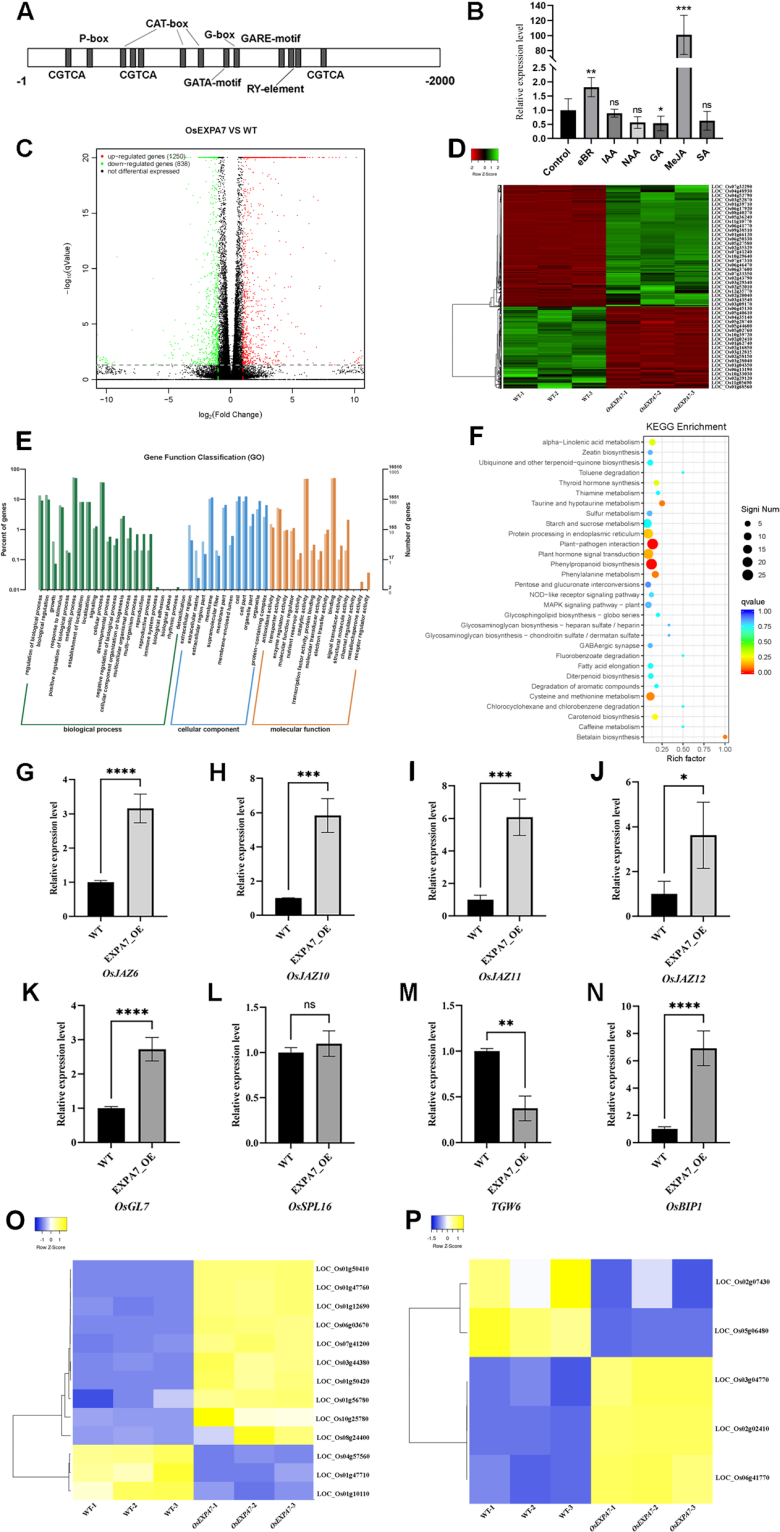
Hormone treatment response and RNA-seq analysis of *OsEXPA7.* **A** Predicted *cis*-acting elements in *OsEXPA7* promoter. **B** Expression levels of *OsEXPA7* in wild-type plants in response to treatment with six different hormones (*n* = 6). **C** Volcano plot of wild-type and *OsEXPA7* OE lines. **D** Heat map of differentially expressed genes (DEGs) between wild-type and *OsEXPA7* OE lines. **E** GO analysis of DEGs. **F** KEGG analysis of DEGs. **G**–**J** Expression levels of *OsJAZ6, OsJAZ10, OsJAZ11,* and *OsJAZ12* in wild-type and OE lines*.* **K**–**N** Expression levels of *OsGL7, OsSPL16, OsTGW6,* and *OsBIP1* in wild-type and OE lines. **O**–**P** Heatmaps of DEGs regulating rice yield and quality

Tools at the PlantCARE database were used to identify *cis*-acting elements in the *OsEXPA7* promoter (Additional file 2: Table S3). Multiple regulatory elements were found in the *OsEXPA7* promoter, such as photoresponse elements, hormone signaling and abiotic stress response elements, meristem expression elements, cell cycle regulatory elements, and seed specific regulatory elements. Among them were multiple CGTCA elements and TGACG elements responsive to Me-JA, as well as GARE elements responsive to GA (Fig. 3A). The presence of these elements suggests that *OsEXPA7* may regulate rice yield traits and quality with the involvement of plant hormone pathways.

To test this hypothesis, wild-type (Nipponbare) rice plants were treated with different plant hormones and the expression levels of *OsEXPA7* were detected by RT-qPCR. The results showed that *OsEXPA7* was significantly up-regulated by BR and Me-JA. In particular, the expression level of *OsEXPA7* was increased by up to 100-fold under Me-JA treatment (Fig. 3B). In contrast, *OsEXPA7* was down-regulated by GA. Treatment with IAA, NAA, and SA did not significantly affect the expression levels of *OsEXPA7*. These results indicate that *OsEXPA7* expression responds to some plant hormones, especially Me-JA.

### *OsEXPA7* Overexpression Affects the Expression of Plant Hormone-Related Genes

To identify the DEGs between wild-type and *OsEXPA7* OE lines, we performed transcriptome sequencing analysis on young panicles during the booting stage. We detected a total of 2088 DEGs (Fig. 3C), of which 1250 were up-regulated and 838 were down-regulated, in the OE lines *vs.* wild type. The DEGs were plotted as heatmaps (Fig. 3D) and subjected to GO enrichment analysis (Fig. 3E). The results showed that the GO terms enriched with DEGs in the OE lines were ‘regulation of biological process’, ‘enzyme regulation’, ‘growth’, ‘metabolic process’ and ‘extracellular region’. These results suggest that *OsEXPA7* is involved in a variety of metabolic processes in plants. In addition, a KEGG database enrichment analysis was conducted to determine which pathways were enriched upon *OsEXPA7* overexpression (Fig. 3F). In the *OsEXPA7* OE lines, the enriched pathways were ‘plant hormone signaling pathway’ and ‘starch and sucrose metabolism’, indicating that *OsEXPA7* may play a role in hormonal pathways and in the regulation of rice quality. Many other genes involved in regulating grain size and rice quality were amongst the DEGs in the *OsEXPA7* OE lines. These genes included those in key QTLs, namely *GL7* (Fig. 3K) and *TGW6* (Fig. 3M), and *OsBIP1* in the seed storage proteins (SSPs) biosynthesis pathway which affects rice quality (Fig. 3N). In addition, the expression level of *BZR1* and *GE* in the BR pathway were also altered. Heatmaps were constructed to illustrate the expression patterns of these DEGs involved in the regulation of grain size and rice quality (Fig. 3O–P).

To further determine which plant hormone pathway were enriched with up-regulated DEGs in the *OsEXPA7* OE lines, we used the RIGW website (http://rice.hzau.edu.cn/cgi-bin/rice_rs2/enrichment) to re-perform the GO and KEGG enrichment analysis for the 1250 up-regulated DEGs. The analysis results show that *OsEXPA7* overexpression led to enrichment of ‘plant hormone signal transduction’, ‘jasmonate ZIM domain proteins’, and ‘phenylalanine ammonia-lyase’ (Additional file 1: Fig. S1A–C). The enrichment of jasmonate ZIM domain proteins was consistent with the results of the hormone treatment experiments, indicating that *OsEXPA7* is likely to be involved in the jasmonate signaling pathway. Therefore, we conducted RT-qPCR analysis to determine the expression levels of genes related to the JA signaling pathway. The results showed that, compared with the control, the OE lines exhibited increased expression levels of *OsJAZ6*, *OsJAZ10*, *OsJAZ11,* and *OsJAZ12* to varying degrees (Fig. 3G–J). *OsJAZs* encode crucial negative regulatory factors in the JA pathway. Therefore, these results indicate that *OsEXPA7* participates in the JA signaling pathway by affecting the expression levels of *OsJAZs*. The above results indicate that *OsEXPA7* overexpression affects the expression of key QTLs as well as genes in the SSPs biosynthesis pathway, BR pathway and JA pathway.

### Haplotype and Population Analyses of *OsEXPA7*

**Fig. 4:**
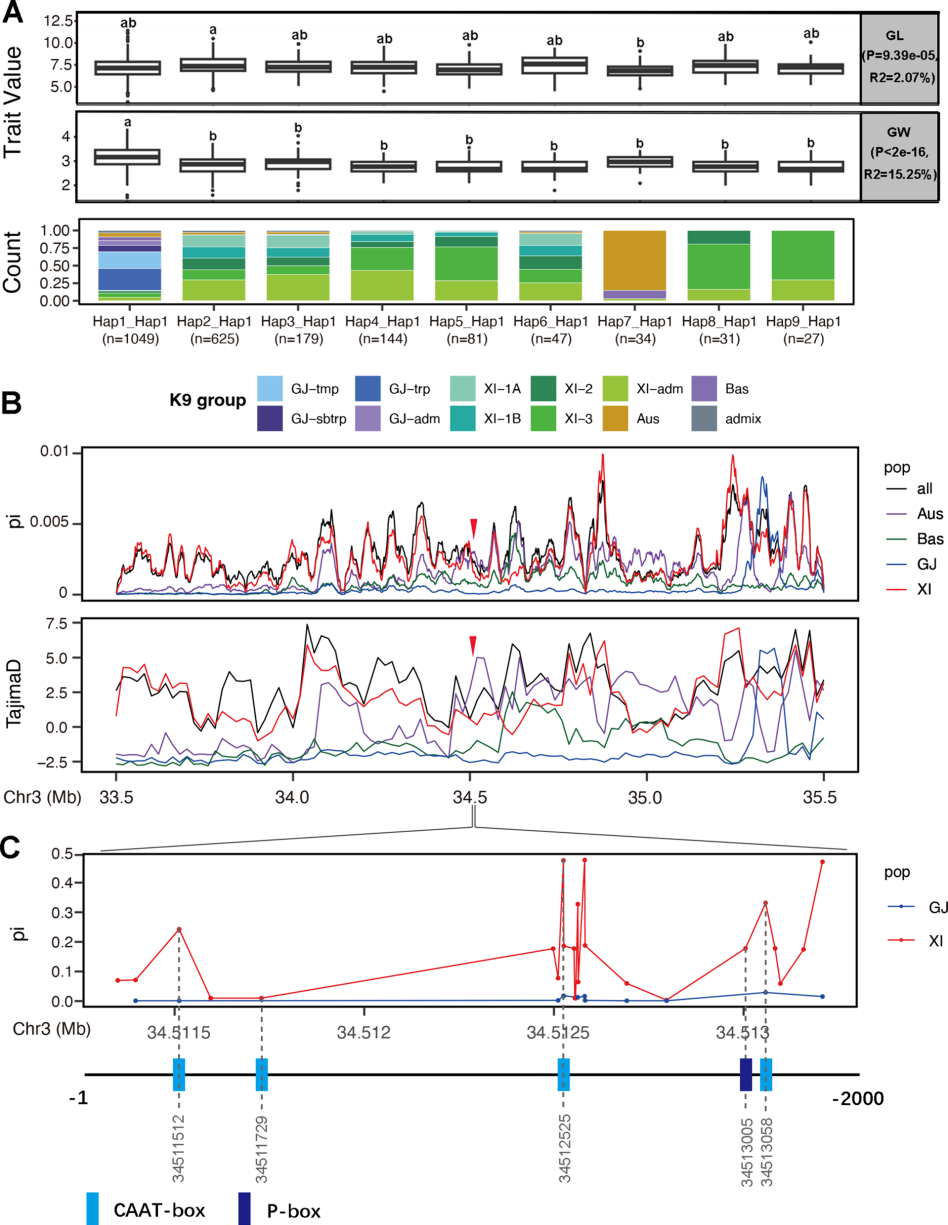
Haplotype analysis and population analysis of *OsEXPA7.* **A** Distribution of grain length and grain width among rice accessions with different *OsEXPA7* haplotypes. **B**, **C** Nucleotide diversity (pi) and Tajima’s D for ∼2-Mb genomic region flanking *OsEXPA7* and nucleotide diversity for upstream 2-Kb promoter region. Red arrow indicates position of *OsEXPA7* and dots indicate the SNP sites among *XI* and *GJ*. Dotted lines represent *cis*-acting elements on the promoter corresponding to candidate functional SNP sites

To clarify the relationship between *OsEXPA7* and the regulation of grain size in rice, we downloaded CDS and promoter gcHaps data for *OsEXPA7* from RFGB and conducted haplotype analysis. We detected nine main haplotypes (≥ 25 rice accessions) and analyzed their relationship with grain length and width. We found that both grain length and grain width were strongly correlated with *OsEXPA7* haplotypes. Hap6_Hap1 had the greatest grain length (8.61 cm ± 1.04 cm), while Hap1_Hap1 had the greatest grain width (3.02 ± 0.42 cm), indicating that these two traits might be affected by different pathways (Fig. 4A). These haplotypes were found to be unevenly distributed among the main population. The haplotypes containing gcHap1 (Hap2_Hap1, Hap3_Hap1, Hap4_Hap1, Hap5_Hap1, Hap6_Hap1, Hap8_Hap1, and Hap9_Hap1) were mostly present in *Xian/Indica (XI),* while Hap1_Hap1 was predominantly present in *Geng/Japonica (GJ).* Interestingly, 80% of Hap7_Hap1 were in the Aus population (Fig. 4A).

To study the evolution of *OsEXPA7*, we conducted a population evolution analysis for the ~ 2 Mb region of *OsEXPA7*. The results showed that there was a significant difference in nucleotide diversity (pi) between the *XI* and *GJ* populations (Fig. 4B). In the *XI* population, the *OsEXPA7* locus was predominantly selected and in the *GJ* population, this locus was secondarily selected (Fig. 4B). Further analysis based on single nucleotide polymorphism (SNP) sites showed that most SNPs were in the promoter region, with four SNPs in the CAAT box in the promoter and one SNP in the P-box (Fig. 4C). The presence of these SNPs may contribute to the differentiation of *OsEXPA7* between *XI* and* GJ.*

## Discussion

### *OsEXPA7* Affects Grain Size by Involving JA Pathway and Key QTLs

**Fig. 5:**
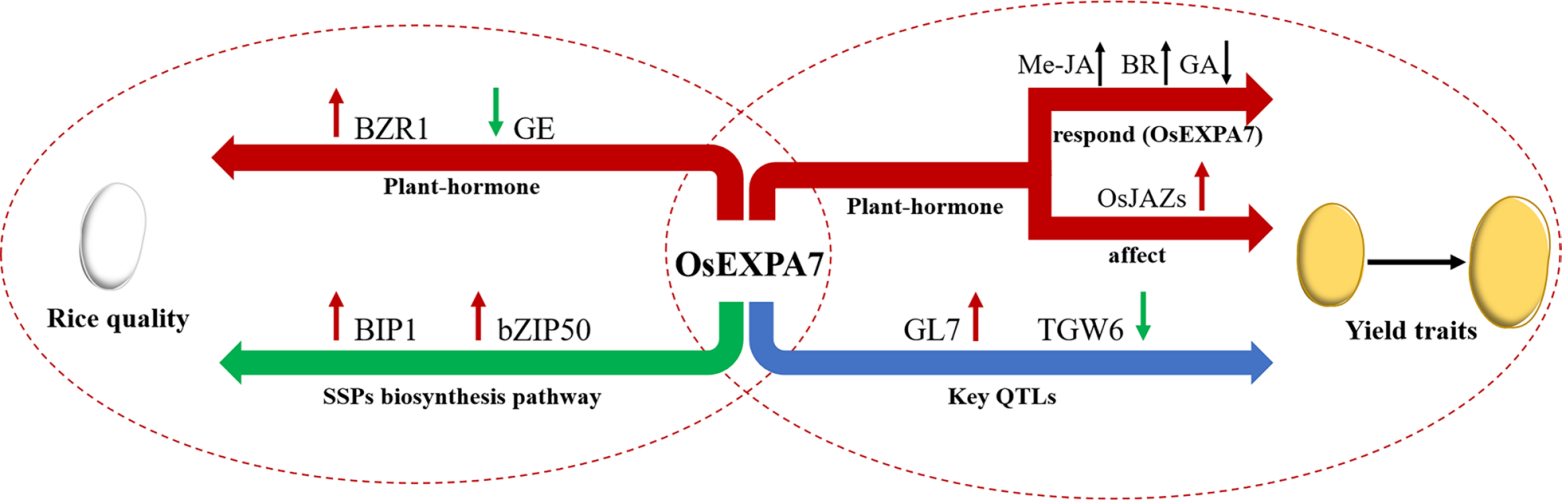
Proposed model of *OsEXPA7* affects rice yield traits and quality

Grain size is an important agronomic trait that determines crop yield. The grain size trait is regulated by various plant hormones, especially JA (Hu et al. 2021). Seed size is inhibited by *COI1* and *MYC2* (and its homologs), *MED25*, and *JAR1* in the JA pathway, but promoted by JAZ proteins (Hu et al. 2021). Another study showed that, compared with the control, the Arabidopsis the JA receptor mutants *coi1-2* and *coi1-8* and the *OsCOI1*-RNAi lines *coi1-13* and *coi1-18* produce significantly larger seeds (Hu et al. 2021). The seed size and hundred-grain weight of the *med25* mutant (pft1-2 and pft1-3) is also significantly increased (Hu et al. 2021). However, the mutant of the JA signaling repressor protein JAZ6 (jaz6-3) exhibits a phenotype with reduced seed length, seed width, and hundred-grain weight (Hu et al. 2021). The JA signaling repressor protein OsJAZ11 is also involved in regulating seed width and weight. In another study, *OsJAZ11-*overexpressing plants showed a 14% increase in seed width and a 30% increase in seed weight, compared with wild type (Mehra et al. 2022). In addition, overexpression lines of *OsJAZ1 (OsTIFY3), OsJAZ5 (OsTIFY9), OsJAZ6 (OsTIFY10a), OsJAZ7 (OsTIFY10b), OsJAZ9 (OsTIFY11a), OsJAZ10 (OsTIFY11b), OsJAZ11 (OsTIFY11c),* and *OsJAZ12 (OsTIFY11d)* all showed growth-enhanced phenotypes, including increased plant height, grain width, and grain weight (Hakata et al. 2017). The results of those studies indicate that *COI1*, *MYC2*, *MED25*, and *JAR1* negatively regulate rice grain size and yield, while JAZ proteins positively regulate rice grain size and yield. In the present study, we found that *OsEXPA7* participates in a variety of hormone pathways. Firstly, in hormone treatment experiments, *OsEXPA7* expression was down-regulated by GA and up-regulated by BR and especially by Me-JA. Secondly, overexpression of *OsEXPA7* up-regulated *OsJAZ6*, *OsJAZ10*, *OsJAZ11*, and *OsJAZ12* in the JA pathway (Fig. 5), but did not affect the expression levels of *COI1*, *MYC2*, *MED25*, and *JAR1*. These results suggest that *OsEXPA7* affects grain size and yield traits with the involvement of several hormone pathways, especially the JA pathway. In the JA signaling pathway, *OsEXPA7* could be activated by JA, and overexpression of *OsEXPA7* can promote *JAZ* expression and inhibit the JA signaling pathway. Therefore, we speculated that *OsEXPA7* plays a signal regulatory role in JA signaling pathway to prevent JA signaling from being uncontrollably amplified indefinitely.

Many QTLs regulating grain shape and size have been identified (Hu et al. 2018a, b). For example, *GL7/GW7/SLG7* is the main QTL controlling grain length, and this region includes a gene encoding a protein containing a TON1 recruitment motif (TRM) to regulate longitudinal cell elongation. The 17.1-kb tandem repeat at the *GL7* locus leads to up-regulation of the *GL7* expression level and down-regulation of negative regulatory factors adjacent to *GL7*, thereby increasing rice grain length and improving rice appearance quality (Wang et al. 2015a, b). Compared with wild type, *GW7-*overexpression lines produced more slender grains and *gw7* mutant plants produced wider grains, indicating that *GW7* positively regulates rice grain length and negatively regulates grain width (Wang et al. 2015a, b). This experimental study found that the expression level of *GL7* gene increased in *OsEXPA7* overexpressing plants (Fig. 5). The increased grain length of the *OsEXPA7* OE line and the unchanged grain width may be a result of the role of *GL7* in panicles. *OsSPL16/GW8* encodes a particle size regulatory factor, namely an SBP-domain transcription factor that inhibits the transcription of *GL7/GW7* (Wang et al. 2015a, b). *OsSPL16* promotes cell proliferation in panicles, resulting in increased grain width (Wang et al., 2012). However, in our study, there were no significant changes in the expression level of *OsSPL16* in the OE plants (Fig. 3L). This finding indicates that *OsEXPA7* regulates *GL7* expression independently of *OsSPL16.*

*TGW6* is a key QTL affecting thousand grain weight, which not only directly controls the length of the endosperm but also indirectly participates in the transportation of carbohydrates from source to sink (Ishimaru et al. 2013). *TGW6* encodes a novel protein with indole-3-acetic acid (IAA)-glucose hydrolase activity, which limits the number of cells and grain length by controlling the supply of IAA to affect the transition of the complex to the cellular stage. The loss of function of *TGW6* increases grain weight and leads to a significant increase in yield traits through its pleiotropic effect on the source organs (Ishimaru et al. 2013). In our study, we found the expression level of *TGW6* was decreased in OE lines. This result suggests that *OsEXPA7* participates in the IAA signaling pathway by regulating the expression level of *TGW6,* thus affecting the source and sink.

### *OsEXPA7* Affects Rice Quality with the Involvement of the BR Pathway and SSPs Biosynthesis Pathway

Plant hormones, including GA, ethylene, abscisic acid, auxin, cytokinin, and BR are important regulators of grain filling, chalkiness, and starch accumulation (Ren et al. 2023). Among them, BR is very important for endosperm development, as it affects grain filling and sugar distribution (Ren et al. 2023). OsBZR1 is a BR signal factor, and inhibition of *OsBZR1* leads to smaller cereal grains and reduced starch accumulation. During grain development, *OsBZR1* directly promotes *Carbon Starved Anther* (CSA) expression, resulting in grain development and sugar accumulation (Zhu et al. 2015). *GE* encodes a cytochrome P450 involved in BR biosynthesis, and it is mainly expressed on the surface of embryonic and endosperm tissues. *GE* mutants have large-embryo and small-endosperm phenotypes (Nagasawa et al. 2013). In rice, BR affects endosperm development by promoting grain filling, which affects rice grain quality. In this study, we found that overexpression of *OsEXPA7* led to increased *BZR1* expression and decreased *GE* expression (Fig. 5), favoring starch accumulation. Interestingly, in our rice quality test, both AC and GC were decreased in the *OsEXPA7* OE lines, indicating that *OsEXPA7* not only involves the BR pathway but also other factors in regulating rice quality.

The biosynthesis of seed storage proteins (SSPs) is a complex process that includes gene transcription, mRNA localization, protein processing in the endoplasmic reticulum (ER), ER to Golgi transfer, and transport of proteins from the Golgi to protein storage vacuoles. Several genes involved in the above steps can affect rice quality (Zhao et al. 2022). *OsBip1* encodes an ER molecular chaperone that plays a role in the translocation of nascent proteins from the ER to the ER lumen. Overexpression of the *BiP1* not only changes the phenotype of seeds and the intracellular structure of endosperm cells, but also reduces the concentration of intracellular storage proteins, starch accumulation, and grain weight (Wakasa et al. 2011). In addition, *OsbZIP50* and *OsBiP1* can activate the unfolded protein response, leading to endosperm softening and shrinkage (Qian et al. 2018; Yang et al. 2022). In our study, the *OsEXPA7* OE lines showed increased expression levels of *OsBip1* and *OsbZIP50* (Fig. 5). This suggests that *OsEXPA7* regulates rice quality by altering the expression of genes in the SSPs biosynthesis pathway.

### Balancing Rice Yield Traits with Quality Traits

Finding the correlation between rice yield and quality is crucial for cultivating high-yielding and high-quality rice varieties. Our results show that *OsEXPA7* positively regulates rice plant height, panicle length, grain length, and thousand grain weight, while negatively regulating rice quality. Further analyses showed that *OsEXPA7* regulates the transcript levels of genes in phytohormone pathways, the expression of key QTLs, and the transcript levels of genes in the SSP biosynthesis pathway, thereby affecting yield traits and rice quality (Fig. 5). These results suggest that the impact of *OsEXPA7* on grain size and quality is not determined by a single pathway but is jointly regulated by a complex network including multiple genes and pathways. Determining the functional characteristics of *OsEXPA7* may help us to understand the factors that affect that balance. In addition, differentiation between the *XI* and *GJ* populations is the mainstream of genetic differentiation in rice, and an understanding of the evolution and kinship of rice is important for the rational use of germplasm resources and variety improvement. The evolutionary analysis of *OsEXPA7* shows that it has nine haplotypes, which are strongly correlated with grain length and width. Multiple SNPs were present within the *cis*-acting elements of the *OsEXPA7* promoter, which may be important for the differentiation of *OsEXPA7* between the *XI* and *GJ* populations. Therefore, in-depth analyses of *OsEXPA7* are important for the cultivation of high-yielding and high-quality rice in a wide range of varieties.

OsEXPA7 positively regulates yield traits and negatively regulates rice quality. *OsEXPA7* expression responds to various phytohormones; it is significantly up-regulated by brassinolide (BR) and methyl jasmonate (Me-JA), but inhibited by gibberellin (GA). *OsEXPA7* affects yield traits and rice quality by regulating the expression of *OsJAZs* in the jasmonate pathway and *BZR1* and *GE* in the BR pathway. OsEXPA7 also affects yield traits and rice quality by regulating the expression of key QTLs (*GL7*, *TGW6*) and genes in the seed storage protein biosynthesis pathway (*BIP1*, *bZIP50*). Red arrows indicate genes up-regulated by *OsEXPA7* and green arrows indicate genes down-regulated by *OsEXPA7.* Black arrows indicate effect of hormones on *OsEXPA7* transcript levels.

## Conclusions

In summary, our results demonstrated that *OsEXPA7* played an important regulatory role in the grain size and quality regulation of rice. *OsEXPA7* positively regulated rice yield traits and negatively regulated rice quality by involving plant hormone pathways, key QTLs and SSPs biosynthesis pathways. This study extended the function of expansins in rice yield and quality traits, and revealed the complex correlation of grain size and quality regulation. The continuous exploration of *OsEXPA7* will contribute to further understand its role in the differentiation of *XI* and *GJ* grain traits and generate high-yielding and high-quality rice varieties.

## Materials and Methods

### Plant Materials and Growing Conditions

Physiological experiments and genetic transformation were conducted using *Oryza sativa* L. spp. *Japonica* cv. Nipponbare. Plant materials were grown in experimental fields in Shanghai (31° 11′ N, 121° 29′ E) and Sanya (18° 14′ N, 109° 31′ E). Standard field management practices were used for transgenic and wild-type plants.

The plant height, panicle length, grain length, grain width, and thousand-grain weight of *OsEXPA7*-overexpressing plants, mutant plants, and wild-type plants were determined using a sample size of 10 per group (*n* = 10). Grain size measurements were conducted using a sample size of 50 seeds from each group.

Tobacco plants (*Nicotiana benthamiana*) were grown in a greenhouse under a 16-h light/8-h dark photoperiod at 26 °C and 50% relative humidity.

### Rice Quality Measurements

Mature rice grains (200 g of each material) of the wild-type (Nipponbare), mutant plants, and *OsEXPA7-*overexpressing plants were dried in an oven at 40 °C for 2 days. After drying, measurements were conducted to determine chalkiness, amylose content, gel consistency, and gelatinization temperature, and each measurement was independently repeated three times (*n* = 3). All data were measured in accordance with the Rice Quality Measurement Standards of the Ministry of Agriculture of the People’s Republic of China (1988). The results are summarized in Table 1.

### Generation of Transgenic Plants

To generate transgenic plants overexpressing *OsEXPA7*, the coding sequence (CDS) of *OsEXPA7* was amplified by PCR from Nipponbare and cloned into the pMD19-T vector. The CDS was then inserted into the pCAMBIA1304 vector to generate the expression vector pCaMV35S:*OsEXPA7*. We generated knock-out mutants of *OsEXPA7 *via CRISPR/Cas9 with the target sequence of 5′-GGTGGTGGTGGGCCGGGGGG-3′. These recombinant construction vectors were transformed into the wild type (Nipponbare) through *Agrobacterium*-mediated transformation. All positively transformed plants were verified by PCR.

### RNA Extraction and Quantitative Real-Time PCR (qRT-PCR)

Total RNA was extracted from rice samples using the FastPure Plant Total RNA Extraction Kit (Vazyme, Nanjing, China). The PrimeScript ™ RT assay kit (Takara, Otsu, Japan) was used to reverse-transcribe RNA into cDNA. The qPCR analyses were performed using the Bio Rad CFX96 real-time fluorescence quantitative PCR system and TB Green Premium Ex Taq II (Takara), with *OsActin* or *OsUBQ5* as internal reference genes. Three biological replicates were analyzed for each sample. The qRT-PCR primers are listed in Additional file 2: Table S2.

### Scanning Electron Microscope Analysis

The outer epidermal cells of mature grain glumes were observed under a scanning electron microscope (TM3000, Hitachi, Tokyo, Japan) at a magnification of 500 times. Cell length and width was measured with ImageJ software (NIH, Bethesda, MD, USA), and the number of cells in the horizontal and vertical directions was calculated (*n* = 10).

### Tissue Expression Profiles

To obtain tissue expression profiles, root and leaf samples were collected from plants at the three-leaf stage, and root, stem, leaf blade, sheath, and young panicle samples were collected from plants at the booting stage. All samples were quickly frozen in liquid nitrogen. Frozen samples (*n* = 3) were ground before extracting RNA for reverse transcription and qPCR experiments (for specific steps, please see "OsEXPA7 Overexpression Affects the Expression of Plant Hormone-Related Genes" section).

### Subcellular Localization of *OsEXPA7*

The CDSs of *OsEXPA7* and *AtPGIP2* (AT5G06870, encoding polygalacturonase-inhibiting protein 2) were amplified and used to construct the fusion vectors 35S::*OsEXPA7-*GFP and 35S::*ATPIPG2-*mcherry, respectively. The fusion vector and control (35S::GFP) were each transformed into *Agrobacterium* GV3101, and then introduced into leaves of 4–5 week old tobacco plants for transient expression. After infiltrating the leaves, the tobacco plants were kept in dark for 24 h and then grown normally for 48 h before detecting fluorescence under a confocal microscope (TCS SP8, Leica Microsystems, Wetzlar, Germany). The cell wall marker in this co-localization analysis was 35S::*ATPIPG2*-mcherry. The average fluorescence intensity of the experimental group and the control group was calculated using the Plot profile function in ImageJ software.

### Plant Hormone Treatment

Two-week-old Nipponbare seedlings were treated with the following six plant hormones: 1 µM brassinolide (BR), 10 µM auxin (IAA), 10 µM naphthylacetic acid (NAA), 100 µM gibberellin (GA), 50 µM methyl jasmonate (Me-JA), or 50 µM salicylic acid (SA) (Lim et al. 2014). After 8 h, the rice roots were collected (0 h as the untreated control) and immediately frozen in liquid nitrogen for total RNA extraction (*n* = 6).

### RNA-Seq Analysis

Total RNA was extracted from young panicles (*n* = 3) of Nipponbare and *OsEXPA7*-overexpressing plants using the FastPure Plant Total RNA Isolation Kit (Vazyme, Nanjing, China). The extracted RNA was used as a template for constructing a cDNA library, which was sequenced by Sangon Biotech (Shanghai, China) using the MGI MGISEQ-2000 platform. The relative gene expression levels were calculated and normalized to the number of fragments per million mapped reads per thousand base transcripts. Differentially expressed genes (DEGs) were annotated and classified based on their presumed or confirmed functions. The GO enrichment analysis was performed using tools at the Gene Ontology Resource website (http://geneontology.org/). The KEGG pathway enrichment analysis was performed using tools at the KEGG database (https://www.genome.jp/kegg/).

### Sequence and Population Evolutionary Analysis of *OsEXPA7*

The CDS haplotypes (gcHaps) and promoter haplotypes (gpHaps) of *OsEXPA7* were together used to analyze two agronomic traits (grain length and grain width) by one-way analysis of variance (ANOVA) and Duncan’s multiple-range test. Differences were considered significant at a probability level of *P* < 0.05. Gene haplotype data were downloaded from the rice functional genomics and breeding (RFGB) database (https://www.rmbreeding.cn). The 3 K phenotype data were obtained from RFGB (https://www.rmbreeding.cn/Phenotype). The dataset used for population evolutionary analysis was downloaded from the rice SNP Seek database (https://snp-seek.irri.org/_download.zul). The 1-Mb region located upstream and downstream of *OsEXPA7* (a 2-Mb region in total) was selected as the target region. VCFtools (v.0.1.15) was used for nucleotide diversity (pi), Tajima’s D, and Fst analyses, and the results were displayed using the R package ggplot2 (v.3.4.0).

For the promoter analysis, a 2000-bp fragment containing the region of − 2000 to − 1 (where the transcription start site of *OsEXPA7* is − 1) was analyzed by PlantCARE (http://bioinformatics.psb.ugent.be/webtools/plantcare/html/) to predict plant *cis*-acting regulatory elements.

### Supplementary Information

**Additional file 1: Fig. S1**. GO and KEGG analyses of up-regulated DEGs identified in RNA-seq analysis of OsEXPA7-OE lines. A–C. KEGG-MAP, GO-term and KEGG-KO of the up-regulated differential genes in OsEXPA7-OE lines.

**Additional file 2: Table S1**. Primers used to confirm transgenic lines. **Table S2** Primers used for the qPCRs. **Table S3** Predicted cis-acting elements of the OsEXPA7 promoter.

## Data Availability

All of the datasets are included within the article and its additional files.

## Abbreviations

EXPA: α-Expansin
OE: Overexpression
KO: Knockout
AC: Amylose content
GC: Gel consistency
PaT: Pasting temperature
JA: Jasmonic acid
Me-JA: Methyl jasmonate
GA: Gibberellin
BR: Brassinolide
SA: Salicylic acid
NAA: Naphthylacetic acid
IAA: Auxin
qRT-PCR: Quantitative real-time PCR
CDS: Coding sequence
QTLs: Quantitative trait loci
ER: Endoplasmic reticulum
SSPs: Seed storage proteins
DEGs: Differentially expressed genes
SNP: Single nucleotide polymorphism
gpHaps: Haplotypes
*GJ*: *Geng/Japonica*
*XI*: *Xian/Indica*
