# Supplementary figures and images for: OsEXPA7 Encoding an Expansin Affects Grain Size and Quality Traits in Rice (Oryza sativa L.)
Source: Rice (N Y). 2024 May 23;17:36. doi: 10.1186/s12284-024-00715-x (PMC11116307; doi:10.1186/s12284-024-00715-x)

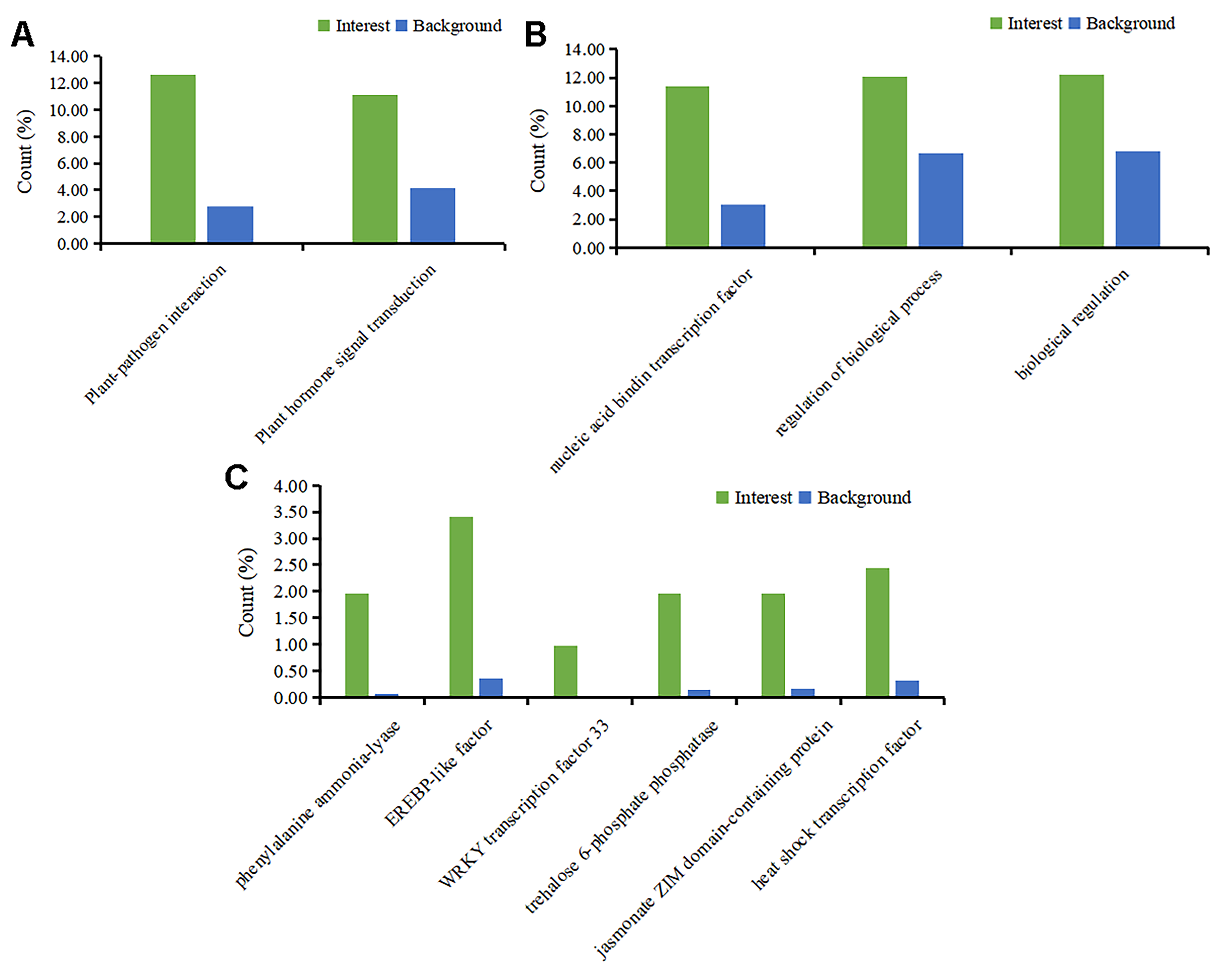

Supplement: Supplementary file 1 — Additional file 1: Fig. S1. GO and KEGG analyses of up-regulated DEGs identified in RNA-seq analysis of OsEXPA7-OE lines. A–C. KEGG-MAP, GO-term and KEGG-KO of the up-regulated differential genes in OsEXPA7-OE lines. [file 12284_2024_715_MOESM1_ESM.tif]
