# Supplementary material for: OsEXPA7 Encoding an Expansin Affects Grain Size and Quality Traits in Rice (Oryza sativa L.)
Source: Rice (N Y). 2024 May 23;17:36. doi: 10.1186/s12284-024-00715-x (PMC11116307; doi:10.1186/s12284-024-00715-x)
Supplement: Supplementary file 2 — Additional file 2: Table S1. Primers used to confirm transgenic lines. Table S2 Primers used for the qPCRs. Table S3 Predicted cis-acting elements of the OsEXPA7 promoter. [file 12284_2024_715_MOESM2_ESM.docx]

**Table S1** Primers used to confirm transgenic lines

| Name | Primers (5’→3’) |
| --- | --- |
| EXPA7-KO-F | CTACTACTACCACGCAGCGG |
| EXPA7-KO-R | CCTGCTCCTCTAACCAAGGC |
| EXPA7-OE-F | ACCTCCACATCCTGGAACG |
| EXPA7-OE-R | CTCATAAATAACGTCATGCATT |

**Table S2** Primers used for the qPCRs

| Name | Primers (5’→3’) | Gene |
| --- | --- | --- |
| qEXPA7-F | TGCCCATGTTCCTCCACATC | LOC_Os03g60720 |
| qEXPA7-R | CGTGATCAGCACCAGGTTGA |  |
| qJAZ6F | GATGGCCAGCAAGAGTTCCT | LOC_Os03g28940 |
| qJAZ6R | GGCAGATCAGCAGCATTTGG |  |
| qJAZ10F | GGATGGTCGTCTTCGAGGAC | LOC_Os03g08330 |
| qJAZ10R | CTTGCGCTTGGCGAAGAACC |  |
| qJAZ11F | GCGCAGCTGACCATCTTCTA | LOC_Os03g08320 |
| qJAZ11R | GCCACGACGATCCTGTTCTT |  |
| qJAZ12F | CCATCTTCTACGACGGGAGG | LOC_Os10g25290 |
| qJAZ12R | ATGGTTCGCTCGTTGTCGTG |  |
| qGL7F | CCATCTCTGTCCTCGACACG | [LOC_Os07g41200](http://rice.uga.edu/cgi-bin/ORF_infopage.cgi?orf=LOC_Os07g41200) |
| qGL7R | CACTGCTCGTCTTGGACTGT |  |
| qGW8F2 | GGAGAGCCCATACTACACGC | LOC_Os08g41940 |
| qGW8R2 | AAGCTGATCTCGCCTTCCTG |  |
| qTGW6F2 | CCCGAAGACTGGCAAATCTGA | LOC_Os06g41850 |
| qTGW6R2 | CGGACCAAAGGGAAGCTCAT |  |
| qBIP1_F1 | TCGCCAATGACCAGGGTAAC | LOC_Os02g02410 |
| qBIP1_R1 | GGGCTTACCGTCCTTGTTCA |  |

**Table S3** Predicted *cis*-acting elements of the *OsEXPA7* promoter

| *Cis*-element | Position | Sequence | Function |
| --- | --- | --- | --- |
| ARE | -415 | AAACCA | *cis*-acting regulatory element essential for the anaerobic induction |
| Box 4 | -1254、-1316 | ATTAAT | part of a conserved DNA module involved in light responsiveness |
| CAT-box | -539、-767、-862 | GCCACT | *cis*-acting regulatory element related to meristem expression |
| CGTCA-motif | -241、-584、-592、-1415 | CGTCA | *cis*-acting regulatory element involved in the MeJA-responsiveness |
| GARE-motif | -1248 | TCTGTTG | gibberellin-responsive element |
| GATA-motif | -923 | GATAGGG | part of a light responsive element |
| G-box | -1052 | CACGAC | *cis*-acting regulatory element involved in light responsiveness |
| MBS | -1392 | CAACTG | MYB binding site involved in drought-inducibility |
| P-box | -379 | CCTTTTG | gibberellin-responsive element |
| RY-element | -1295、-1299 | CATGCATG | *cis*-acting regulatory element involved in seed-specific regulation |
| TGACG-motif | -241、-592、-584、+1415 | TGACG | *cis*-acting regulatory element involved in the MeJA-responsiveness |
